# Endocannabinoid oxygenation by prostaglandin H synthase-2: Chemistry and biology

**DOI:** 10.1016/j.jbc.2025.110870

**Published:** 2025-10-28

**Authors:** Carol A. Rouzer, Lawrence J. Marnett

**Affiliations:** Departments of Biochemistry, Chemistry, and Pharmacology, Vanderbilt Institute of Chemical Biology, Vanderbilt-Ingram Cancer Center, Vanderbilt University School of Medicine Basic Sciences, Nashville Tennessee, USA

**Keywords:** endocannabinoid, 2-arachidonoylglycerol, cyclooxygenase, prostaglandin, prostaglandin glyceryl ester, inflammation, macrophage, neuron

## Abstract

Prostaglandin (PG) endoperoxide synthases (PGHS)-1 and -2 oxygenate arachidonic acid to PGH_2_, the precursor to PGs and thromboxane. PGHS-2 also oxygenates ester and amide substrates more efficiently than PGHS-1. Foremost among these neutral substrates are the endocannabinoids, arachidonoylethanolamide and 2-arachidonoylglycerol (2-AG). 2-AG is the most abundant endocannabinoid and a full agonist for both cannabinoid receptors, CB1 and CB2. PGHS-2-mediated oxygenation of 2-AG ultimately produces PG glycerol esters, which exhibit biological activities largely independent of classical endocannabinoid and PG receptors. This article reviews the mechanism of PGHS-2 oxygenation of 2-AG, the kinetic and structural basis for its biochemical regulation, the occurrence of 2-AG oxygenation in cells and *in vivo*, and the biological impact of the pathway. Gaps in our knowledge and challenges to a fuller understanding of the impact of PGHS-2 oxygenation of 2-AG are presented.

Oxygenation of arachidonic acid (AA) by prostaglandin (PG) H synthase (PGHS) enzymes—also known as cyclooxygenases (COX-1 and COX-2)—ultimately produces PGs E_2_, D_2_, F_2α_, I_2_, and thromboxane A_2_ (TxA_2_). They were the first oxygenated polyunsaturated fatty acids isolated in the animal kingdom, and they exert a broad range of physiological and pathophysiological activities. Inhibition of PG synthesis is a key mechanism of action of nonsteroidal anti-inflammatory drugs (NSAIDs), and extensive efforts are underway to develop agonists or antagonists of individual PG receptors. Although PGs were identified over 60 years ago, the study of their functions remains an area of active investigation.

PGHS has a pair of catalytic activities—a heme-dependent peroxidase that activates a fatty acid COX. The two activities conspire to oxygenate AA into the hydroperoxy endoperoxide, PGG_2_, and then reduce it to the hydroxy endoperoxide, PGH_2_ (Fig. 1). There are two PGHS enzymes (PGHS-1 and PGHS-2), each comprising two identical 70 kDa subunits. Each subunit has an epidermal growth factor domain near the N terminus, a membrane-binding domain, and a catalytic domain (Fig. 2*A*). Within the catalytic domain is a binding site for the heme prosthetic group that plays a critical role in the peroxidase reaction. The peroxidase active site is separated from the COX active site by a strand of polypeptide containing the proximal ligand to the heme (His-388) and the catalytic tyrosine (Tyr-385) (Fig. 2*A*). Tyr-385 is oxidized to a tyrosyl radical by a higher oxidation state of the peroxidase (1). The tyrosyl radical initiates the COX reaction by removing the 13-*pro*(*S*)-hydrogen of AA (Fig. 2*B*) (2). The need for peroxidase-mediated tyrosyl radical activation necessitates that some amount of hydroperoxide is present in a cell for the COX reaction to occur. In addition, the PGHS enzymes undergo peroxide-dependent suicide inactivation during catalysis. Thus, cellular peroxide tone plays an important role in the modulation of PGHS activity (3).

**Figure 1:**
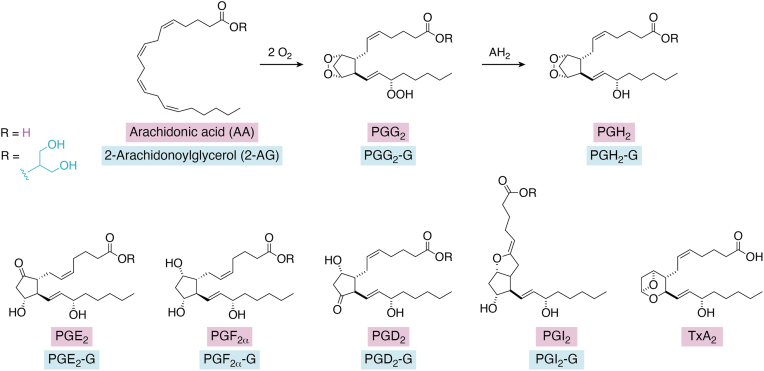
**Formation of PGs and Tx from AA or PG-Gs from 2-AG.** Upper*,* conversion of AA (R = H) to PGG_2_ and PGH_2_, or 2-AG (R = C_3_H_7_O_2_) to PGG_2_-G and PGH_2_-G. Lower*,* products of the enzymatic conversion of PGH_2_ or PGH_2_-G. Note that PGH_2_-G is a poor substrate for thromboxane synthase, which produces TxA_2_ from PGH_2_. AA, arachidonic acid; AG, arachidonoylglycerol; PG, prostaglandin; PG-G, prostaglandin glyceryl ester; PGH_2,_ prostaglandin H_2_; Tx, thromboxane.

**Figure 2:**
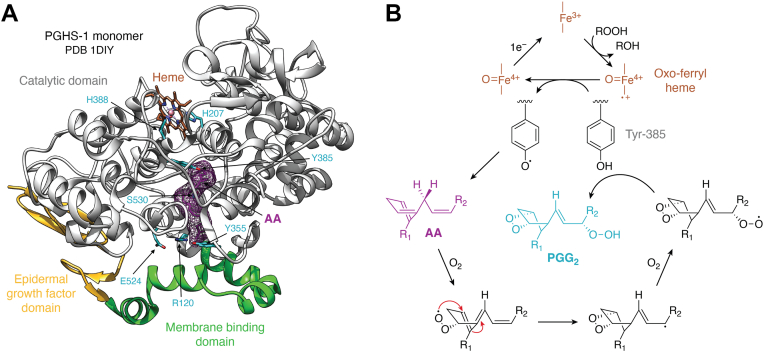
**PGHS structural features and catalytic cycle.***A,* X-ray crystal structure of one monomer of PGHS-1 as derived from PDB 1DIY. The EGF domain (*gold*), membrane-binding domain (*green*), and catalytic domain (*gray*) are indicated. The heme prosthetic group is shown in *sienna*, and the bound AA is shown in plum mesh. Side chains of key residues that are highlighted in *cyan* include the proximal heme ligand (His-388), the distal heme ligand (*via* a coordinating water molecule), His-207, Tyr-385, which initiates the cyclooxygenase reaction, Ser-530 (located behind the AA mesh), which is the site of aspirin acetylation, and Glu-524, Tyr-355, and Arg-120, which form a constriction at the opening of the active site channel. *B,* in the peroxidase active site, reduction of a substrate hydroperoxide (ROOH) to the corresponding alcohol creates the oxoferryl heme, which oxidizes Tyr-385 in the cyclo-oxygenase active site to a tyrosyl radical. The radical abstracts a hydrogen atom from the 13-position of AA, which subsequently reacts with two oxygen molecules to form PGG_2_. The final step in PGG_2_ formation regenerates the tyrosyl radical. PGG_2_ can then serve as a substrate for the peroxidase reaction. AA, arachidonic acid; EGF, epidermal growth factor; PDB, Protein Data Bank; PGHS, prostaglandin endoperoxide synthase.

Differential regulation of the two PGHS genes was the major focus of attention following the discovery of PGHS-2. The similarity in overall structure between the two proteins, especially within the AA-binding site, implied that their biochemical functions are similar. However, studies by the Smith lab of the role of individual residues near the active site entrance raised the possibility that there may be subtle functional differences. Mutation of Arg-120 of PGHS-1 to Gln increased the *K*_*M*_ for AA nearly 1000-fold indicating the importance of an ionic bond between the carboxylic acid of the substrate and the enzyme at that position (4). In contrast, the same mutation in PGHS-2 had no effect on the *K*_*M*_ for AA, implying that hydrophobic or nonionic polar interactions with the substrate are more important than ionic interactions (5).

An important implication of these observations is that PGHS-2 may bind neutral substrates or inhibitors more effectively than PGHS-1. Indeed, arachidonoylethanolamide (AEA, anandamide) had been reported to be oxidized by PGHS-2 but with a *k*_cat_/*K*_*M*_ 500-fold lower than that for AA (6). Furthermore, neutral derivatives of carboxylic acid inhibitors, such as indomethacin, were discovered to be highly selective inhibitors of PGHS-2 compared with PGHS-1 (7, 8). These findings preceded the discovery that 2-arachidonoylglycerol (2-AG) is a selective substrate for PGHS-2 and that, in contrast to AEA, the *k*_cat_/*K*_*M*_ for 2-AG is the same as the *k*_cat_/*K*_*M*_ for AA (9). Comparison of a series of arachidonoyl esters indicated that 2-AG is the preferred substrate. Its isomerization product, 1(3)-AG, is 40% as efficient as 2-AG and similar to arachidonoylglycol as a substrate for PGHS-2. The presence of a hydroxyl group is important, as demonstrated by the low activity of PGHS-2 toward methyl arachidonate, ethyl arachidonate, and β-methoxyethyl arachidonate (9). 1,2-Diarachidonoylglycerol is also a poor substrate for PGHS-2.

The products of 2-AG oxygenation by PGHS-2 were identified as the glyceryl ester of prostaglandin H_2_ (PGH_2_-G), along with smaller amounts of the hydroxy fatty acylglycerols 11- and 15-hydroxyeicosatetraenoic acid glyceryl esters, mirroring the free acid products derived from the oxygenation of AA. In cells, PGH_2_-G is converted to other PG glycerol esters. These are referred to here collectively as PG-Gs and include, specifically, PGE_2_-G, PGD_2_-G, PGF_2α_-G, and PGI_2_-G (Fig. 1*B*), depending on the availability of the relevant isomerases (9, 10). The percentage conversions of PGH_2_-G to the different classes of products are similar to those observed with PGH_2_ produced from AA.

Arachidonoyl amino acids and amines are also substrates for PGHS-2. *N*-Arachidonoyl glycine is the most selective of all the PGHS-2 substrates, demonstrating no oxidation by PGHS-1 and an extent of oxidation by PGHS-2 ∼40% of that of AA (11). *N*-Arachidonoyl alanine and *N*-arachidonoyl-γ-aminobutyric acid are oxidized ∼30% and 20% as effectively as AA, respectively (11, 12). *N*-Arachidonoyl vanilloids are less efficiently oxygenated by PGHS-2 than 2-AG. *N*-Arachidonoyl dopamine, *N*-arachidonoyl *O*-(3-methyl)dopamine, and *N*-arachidonoyl arvanil are each oxygenated less than 10% of the extent of AA (12). A profile of PG products similar to those observed from 2-AG is observed from AEA, and investigations have been conducted on their formation and biological activities (13, 14, 15, 16). They will not be the focus of the current review.

An interesting variant on this theme is the selective oxygenation of 2-arachidonoyl lysophospholipids. Gross *et al.* discovered the existence of a membrane-associated, Ca^2+^-independent phospholipase A2γ that selectively hydrolyzes *sn*-1 fatty acids from phospholipids containing polyunsaturated fatty acids at the *sn*-2 position, yielding the 2-acyl-lysophospholipids (17). Arachidonoyl lysophosphatidylcholine and arachidonoyl lysophosphatidylethanolamine are then oxidized by PGHS-2 to PG and hydroxy fatty acyl lysophospholipids. These compounds are detectable in intact cells, mouse liver, and human myocardium (18). The levels of the PG-lysophospholipids in mouse liver are markedly decreased in independent phospholipase A2γ knockout animals (18).

Vecchio and Malkowski (19) determined the crystal structure of PGHS-2 in complex with 1(3)-AG. Note that isomerization of 2-AG to 1(3)-AG in aqueous solution precluded obtaining a structure containing 2-AG. The endocannabinoid has a similar binding pose to that of AA in the COX active site, although there are subtle differences in the distances and orientations of the 13-*pro*(*S*) hydrogens to the catalytic Tyr-385. Leu-531 is a critical residue in determining the ability of PGHS-2 to oxidize 2-AG. The glycerol ester moiety of 2-AG binds adjacent to Leu-531 and forces it to rotate away from the active site (19). Differences in the sequence and local structure of adjacent residues in the vicinity of residues 115 to 125 between PGHS-2 and PGHS-1 may facilitate the altered conformation of Leu-531 in PGHS-2 (20). The differences in the orientation of the 13-*pro*(*S*) hydrogens to the catalytic Tyr-385 may account for the sensitivity of 2-AG oxygenation by PGHS-2 to allosteric inhibition by certain NSAID derivatives as discussed later (21, 22, 23). It may also explain the observation that PGHS-2 requires a higher peroxide tone when oxidizing 2-AG than when it is oxidizing AA, which results in greater sensitivity of 2-AG oxygenation to agents that scavenge hydroperoxides (24).

The discovery of the ability of PGHS-2 to oxygenate neutral derivatives of AA, particularly the endocannabinoids, has naturally led to many important questions. What regulates the oxygenation of 2-AG by PGHS-2? What is the impact of oxygenation on endocannabinoid signaling? Where, and under what circumstances, does PGHS-2-dependent endocannabinoid oxygenation occur? Do the resulting products have biological activity? In this review, we summarize the current knowledge surrounding these questions with emphasis on the oxygenation of 2-AG to PG-Gs.

## Impact of PGHS-2 allostery on 2-AG oxygenation

### Discovery of PGHS allosteric regulation

Although the PGHS enzymes are homodimeric proteins, evidence emerged over 40 years ago that the two subunits exhibit distinct activities. The first observations came from Kulmacz and Lands (25), who showed that maximal enzyme activity of PGHS-1 is achieved upon binding of just one heme molecule per dimer. Similarly, they demonstrated that the slow, tight-binding inhibitors indomethacin, meclofenamic acid, and flurbiprofen achieve maximal inhibition at a ratio of one molecule per dimer (26). These findings suggest that only one monomer binds heme and is catalytically active and that inhibitor binding to only one monomer is sufficient to block enzyme activity.

Ten years later, the Swinney laboratory published data suggesting that PGHS-1, but not PGHS-2, is an allosteric enzyme that exhibits positive cooperativity with respect to AA (27). Mutation of Tyr-355 to Phe in PGHS-2 conveyed positive cooperativity to that isoform, suggesting an important role for this critical constriction site residue at the entrance to the active site (28). The observed cooperativity in PGHS-1 could, however, be explained by the requirement of PGHS for peroxide-dependent activation, which is more rapid and complete in the presence of higher concentrations of substrate. The finding by Kulmacz and Wang (29) that PGHS-1 requires higher levels of hydroperoxide than PGHS-2 would be consistent with seeing the “cooperativity” only in the PGHS-1 isoform.

More detailed insight into the allosteric behavior of the PGHS enzymes was provided by the Smith laboratory in a series of publications beginning in 2006. The first of these employed the construction of heterodimers of PGHS-2 comprising one native enzyme subunit and one subunit bearing a site-directed mutation (30). Incorporation of an inactivating G533A mutation in one subunit resulted in a heterodimer (native/G533A) that retained the same specific activity as the native enzyme homodimer. Similarly, a native/R120Q heterodimer contained two catalytically active subunits, whereas only the native subunit retained the ability to bind and be inhibited by flurbiprofen. This heterodimer exhibited similar sensitivity to flurbiprofen inhibition as the native homodimer. These findings provided further support for the concepts first outlined by Kulmacz and Lands that ligand binding to only one subunit is required for catalysis and/or inhibition.

The Smith lab revisited the binding of heme to PGHS-2 using both enzyme activity and spectroscopic methods. They concluded that the enzyme has only one high-affinity heme-binding site with a *K*_*D*_ value between 10 and 100 nM. This led them to propose that the subunit with bound heme is the catalytic subunit (*E*_cat_), whereas the other serves an allosteric function (*E*_allo_) (31). Experiments employing PGHS-2 heterodimers provided additional support for this hypothesis (32). Further work demonstrated that a number of nonsubstrate fatty acids could serve as allosteric activators to stimulate the activity of PGHS-2 but not PGHS-1. Palmitic acid was the most efficacious of the fatty acids tested, and its stimulatory effect was attributed to a lowering of the enzyme's *K*_*M*_ for AA (33).

Application of their model to inhibitors led to the conclusion that some (celecoxib, diclofenac, indomethacin, and rofecoxib) block enzyme activity by binding competitively to *E*_cat_, whereas others (naproxen and flurbiprofen) inhibit noncompetitively by binding to *E*_allo_ (31). Aspirin, which blocks enzyme activity by covalently acetylating Ser-530, was demonstrated to react in only one subunit of PGHS-2. When compared with native PGHS-2, the acetylated enzyme produced much lower quantities of PGH_2_ and higher quantities of the side product, 15-hydroperoxyeicosatetraenoic acid (15-HPETE). Binding of diclofenac to acetylated PGHS-2 blocked the production of PGH_2_ but not 15-HPETE (32, 34). This led the investigators to conclude that the unacetylated subunit produces PGH_2_, whereas the acetylated subunit produces 15-HPETE; however, this theoretically could only happen if heme is present in both subunits. Notably, aspirin had previously been shown to acetylate only one subunit of PGHS-1, and the presence of heme in the enzyme was required (35). This study did not address whether one or two hemes were bound to the enzyme dimer. Thus, we do not know which subunit was acetylated if only one subunit contained heme (35).

As noted above, the finding that full activity of PGHS requires only a single heme monomer per dimer has led to the assumption that PGHS in solution contains only one heme. Consequently, the monomer to which it is bound must serve as *E*_cat_, thereby locking each subunit into its respective role. Furthermore, data have been reported suggesting a pre-existing asymmetry of the enzyme that could be explained by asymmetric heme binding (32), although most of these experiments have employed PGHS-2 heterodimers, which are, *a priori*, asymmetric proteins. Conversely, an investigation of the location and identity of tyrosyl radicals generated in PGHS-2 during catalysis in solution demonstrated the presence of radicals at Tyr-385 in both subunits (36), suggesting that both subunits must contain the heme required to generate them. Moreover, in the numerous published crystal structures of the holoenzyme, heme is visualized in both subunits. Thus, there is some reason to question whether the enzyme in solution contains one or two hemes per dimer; however, it is not necessary to posit heme binding in only one subunit to explain the allosteric behavior of PGHS.

### Structural basis for allostery

**Figure 3:**
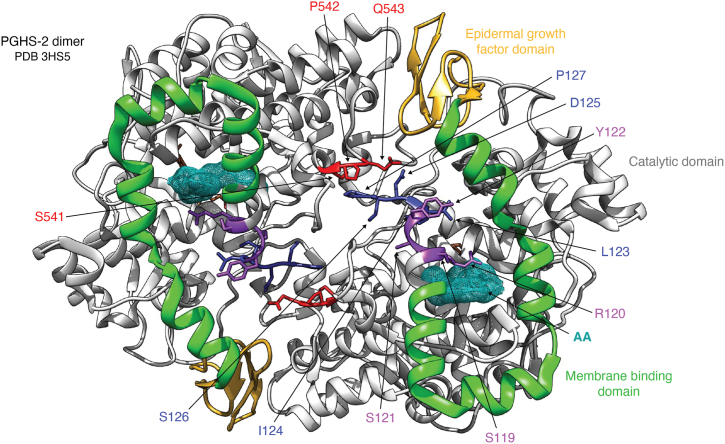
**Structural basis of PGHS-2 allosteric regulation.** A model of the dimeric structure of PGHS-2 derived from PDB 3HS5 looking up into the active site through the membrane-binding domain highlights structures believed to be involved in allosteric regulation. The epidermal growth factor domain (*goldenrod*), membrane-binding domain (*green*), and catalytic domain (*light gray*) are indicated. Bound AA (*turquoise mesh*) and heme (*sienna*, located above the AA) are included. A loop containing residues Ser-541, Pro-542, and Gln-543 (*red*) on one subunit interacts with a loop containing residues Leu-123, Ile-124, Asp-125, Ser-126, and Pro-127 (*dark blue*) on the other subunit. Paired mutations at positions 541 and 127 or at positions 126 and 543 to cysteine lead to crosslinking of the two subunits that is inhibited by flurbiprofen (33). The loop containing Ser-119, Arg-120, Ser-121, and Tyr-122 (*dark magenta*) is the site of the S121P mutation that was shown to disrupt allosteric modulation. This loop also contains Arg-120, which plays a role in stabilizing AA binding at the active site constriction (38). AA, arachidonic acid; PDB, Protein Data Bank; PGHS, prostaglandin endoperoxide synthase.

As noted above, attempts to elucidate the structural basis for PGHS allostery were hampered by the fact that in the numerous published crystal structures of the holoenzyme, heme is present in both subunits. Similarly, complexes containing inhibitors invariably demonstrate ligands in both subunits with no obvious structural differences between them. A possible explanation for this is that enzyme conformations that are stable for crystal formation are symmetrical ones in which both heme and active sites are filled. To overcome this difficulty, the Smith group crystallized PGHS-1 and -2 proteins with a number of inhibitors under various conditions designed to favor binding in only one subunit. These efforts were met with limited success, but they did enable the identification of a loop of residues (123–127) that exhibits distinct conformations in bound *versus* unbound subunits (37). Notably, this loop is located at the dimer interface, where it interacts with residues 541 to 543 of the opposing subunit (Fig. 3). Prior work using cross-linking techniques had identified these two regions of the enzyme as being involved in ligand-mediated subunit interactions (33). Additional studies of an S121P mutant homodimer of PGHS-2 demonstrated higher activity and resistance to palmitic acid–mediated allosteric regulation as compared with that of the native enzyme. A crystal structure of the S121P enzyme revealed displacement of residues 119 to 122, and cross-linking studies demonstrated that the S121P mutation was prone to undergo spontaneous crosslinking of the two subunits in the absence of ligands. The results led to the hypothesis that the 120 to 129 residue loop tonically inhibits *E*_cat_ and that movement of the loop in response to allosteric activators releases the inhibition. The S121P mutation achieves that same movement in the absence of an allosteric ligand (38). More recently, exploration of the role of this region in allosteric activation was carried out by Dong and Malkowski (39, 40), who used ^19^F-NMR to demonstrate multiple conformations of PGHS-2 and the modulation of these conformations upon binding of allosteric modulators.

### Impact of allostery on the competitive kinetics of AA *versus* 2-AG

Although AA and 2-AG are oxygenated with similar catalytic efficiency by PGHS-2 *in vitro*, when both substrates are present in intact cells, the ratio of PGs to PG-Gs synthesized far exceeds the ratio of AA to 2-AG. This led our laboratory to explore the interaction of the two substrates at *E*_cat_ and *E*_allo_ of PGHS-2 when presented simultaneously to the purified enzyme (41). Kinetic studies were carried out using 5-phenyl-4-pentenyl-hydroperoxide to ensure rapid and efficient enzyme activation and 10 s incubation times to minimize the effects of substrate consumption and enzyme inactivation. The data were analyzed using an unbiased systems-based mechanistic model that provided the most highly probable *K*_*D*_ values for all possible complexes and the *k*_cat_ values for all possible catalytically competent assemblies. The results suggested that AA binds to *E*_cat_ with greater affinity than to *E*_allo_, but binding to *E*_cat_ greatly increases *E*_allo_ affinity. The presence of AA in *E*_allo_ has little effect on its own oxygenation, but it suppresses oxygenation of 2-AG. Similarly, 2-AG binds with greater affinity to *E*_cat_ than *E*_allo_, but the affinity for *E*_allo_ increases once *E*_cat_ is bound. Experimental data revealed that 2-AG exhibits substrate inhibition, and this was incorporated into the model. Thus, 2-AG binding in *E*_allo_ inhibits 2-AG oxygenation, but it was found to stimulate AA oxygenation. These findings could help to explain why AA is preferentially oxygenated in cells.

The mathematical model for the interaction of 2-AG with AA proposed by our laboratory differs significantly from that of Dong *et al.*, which posits that fatty acid binding occurs to *E*_allo_ first, followed by binding to *E*_cat_. They also reported that 2-AG does not bind to *E*_allo_ with appreciable affinity or exert any allosteric effect on enzyme activity (31, 33, 42). Notably, the model developed by Dong *et al.* was based on experiments using very high concentrations of enzyme and high enzyme-to-substrate ratios. They also did not control for enzyme activation or inactivation in those studies. Thus, there are fundamental differences in the approaches used to generate each model. It is important to note that the number of potential interactions of substrates and allosteric modulators with PGHS is large, complicating any attempt at kinetic analysis. Our reported findings represent the calculated most probable set of parameters based on our model and initial assumptions, but this is, by no means, the only possible set. Confirmation of either model requires further investigation.

### Role of allostery in substrate-selective inhibition of 2-AG oxygenation

**Figure 4:**
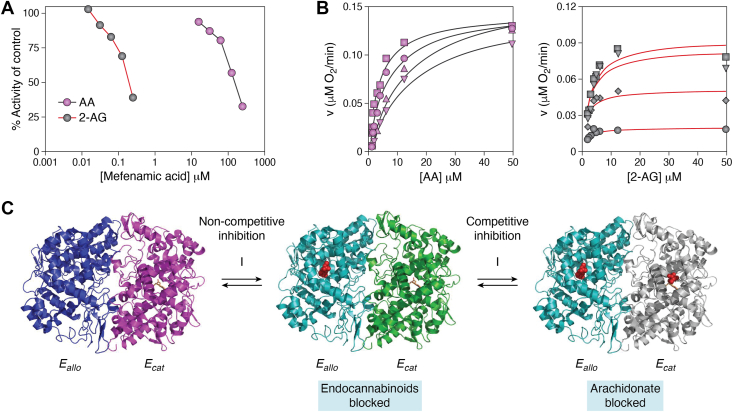
**Substrate-selective inhibition of PGHS-2 oxygenation of 2-AG and AA by mefenamic acid.***A,* comparison of IC_50_'s for mefenamate with 2-AG and AA. *B,* comparison of kinetics of inhibition by mefenamate for AA (*left*) and 2-AG (*right*). Reproduced under the *ACS AuthorChoice/Editors' Choice* usage agreement from (21). Copyright 2009 American Chemical Society. *C,* proposed mechanism for substrate selectivity of inhibition of 2-AG oxygenation. *Far left*—binding of heme to one subunit (*pink*) establishes this subunit as *E*_cat_ and the other as *E*_allo_ (*navy blue*). *Center*—when a substrate-selective inhibitor (*red spheres*) binds to *E*_allo_ with high affinity, a conformational change (*turquoise*) occurs that renders *E*_cat_ (*green*) unable to oxygenate 2-AG but retains activity with AA. *Far right*—lower affinity binding of the inhibitor to *E*_cat_ (*gray*) competitively blocks AA oxygenation as well. AA, arachidonic acid; 2-AG, 2-arachidonoylglycerol; PGHS, prostaglandin endoperoxide synthase.

In 2009, our laboratory reported that ibuprofen and mefenamic acid, which are both weak competitive inhibitors of AA oxygenation by PGHS-2, are potent noncompetitive inhibitors of 2-AG oxygenation (Fig. 4*A*). In contrast, the slow, tight-binding inhibitor indomethacin exhibits equal potency against oxygenation of both substrates. These findings led to the hypothesis that the observed substrate-selective inhibition of 2-AG oxygenation results from high affinity binding of the inhibitor to *E*_*a*llo_, which then leads to a conformational change in the enzyme that blocks 2-AG oxygenation but not AA oxygenation (Fig. 4*B*). To inhibit the oxygenation of AA, the inhibitor must also bind competitively to *E*_cat_, a site for which it has lower affinity (21).

**Figure 5:**
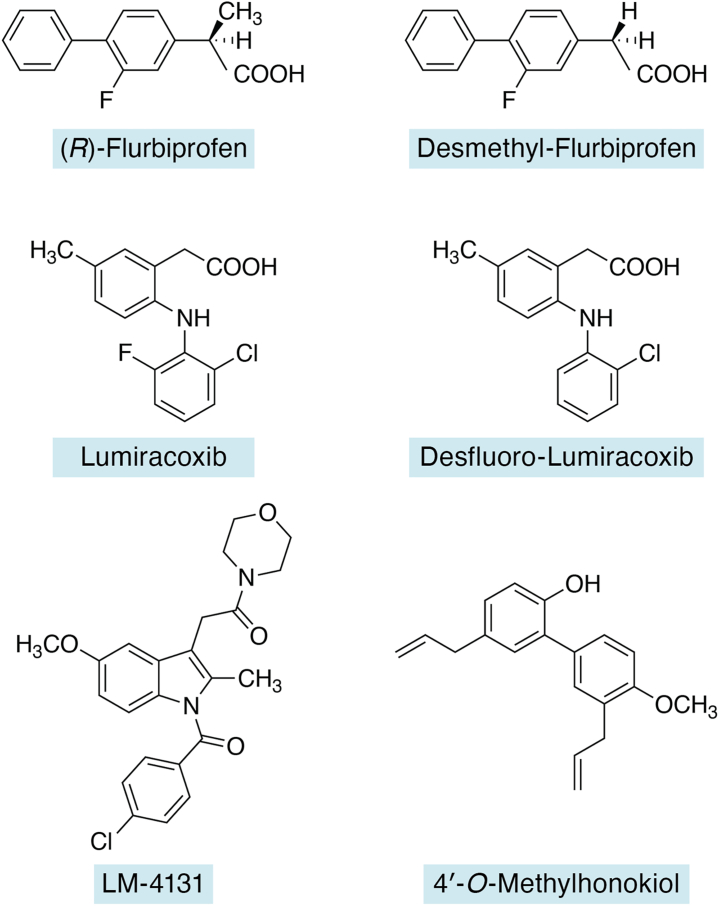
**Structures of substrate-selective inhibitors from different structural scaffolds**.

Subsequent studies demonstrated that the (*R*)-enantiomers of ibuprofen, naproxen, and flurbiprofen are substrate-selective inhibitors of 2-AG oxygenation by PGHS-2. The findings were unexpected because it had been assumed that the positioning of the α-methyl group of these molecules would preclude binding to the PGHS active site due to a clash with Tyr-355. Crystal structures demonstrated that the (*R*)-enantiomers bind in the PGHS-2 active site in a pose very similar to those of their corresponding (*S*)-enantiomers, although a repositioning of Tyr-355 and Arg-120 is required to accommodate the α-methyl group (43). These findings led to the synthesis of a series of desmethyl, dimethyl, and cyclopropyl analogs of flurbiprofen, fenoprofen, naproxen, ketoprofen, and ibuprofen in a search for more selective and potent inhibitors of 2-AG oxygenation (22). In general, the desmethyl analogs are the most active in any series, and flurbiprofen is the most potent scaffold (Fig. 5). *In vivo*, desmethyl–flurbiprofen exhibits a relatively short plasma half-life (2–3 h). (*R*)-flurbiprofen is metabolized more slowly, but substantial conversion to the (*S*)-enantiomer is observed (22). Compared with (*R*)-flurbiprofen, the desmethyl analog is more potent but less selective. In a separate study, Karlsson and Fowler (44) reported that some physiological metabolites of flurbiprofen and ibuprofen exhibit substrate-selective inhibition of 2-AG oxygenation; however, they concluded that these activities likely do not contribute significantly to the overall pharmacologic effects of the drugs *in vivo*.

Lumiracoxib, a highly potent PGHS-2-selective inhibitor *in vivo*, is a relatively weak, time-dependent inhibitor *in vitro* that also exhibits substrate-selective inhibition of 2-AG oxygenation. An investigation of the structural determinants of substrate selectivity of lumiracoxib determined that the hydrogen-bonding potential of substituents at the *ortho* positions of the aniline ring determines potency and substrate selectivity. A 5′-methyl group on the phenylacetic acid ring in the presence of a single *ortho* substituent on the aniline ring, as observed in desfluorolumiracoxib, yields the highest potency among the analogs tested (23).

All the identified substrate-selective inhibitors discussed previously are either known NSAIDs or derivatives of known NSAIDs. However, a more recent search to identify novel scaffolds displaying this activity led to the discovery of an analog of harmaline, a naturally occurring tricyclic indole (45). A crystal structure of the active analog complexed with PGHS-2 revealed a binding mode completely dependent on nonpolar interactions that require displacement of Leu-531 toward the dimer interface. No further work on this compound has been reported. Similarly, the selective CB2 receptor antagonist 4′-*O*-methylhonokiol and a number of its analogs display substrate-selective inhibition of 2-AG oxygenation *in vitro* and in intact lipopolysaccharide (LPS)-pretreated RAW264.7 cells that express PGHS-2. In *in vivo* studies of LPS-challenged mice, 4′-*O*-methylhonokiol treatment increased 2-AG levels in the brain, but PG-G levels were not reported (46).

In their studies focusing on the fenamic acid series of inhibitors, Orlando and Malkowski (47) confirmed rapid and reversible substrate-selective inhibition of 2-AG oxygenation by mefenamic acid, tolfenamic acid, and flufenamic acid. In contrast, meclofenamic acid was found to be a potent time-dependent inhibitor of both 2-AG and AA oxygenation. Crystal structures of the fenamic acids revealed a similar binding pose for all that places the carboxylic acid of each close to the catalytic Tyr-385 residue (47). Spectroscopic studies demonstrated rapid quenching of the tyrosyl radical of PGHS-2 by the three substrate-selective inhibitors, whereas quenching by meclofenamic acid was time dependent. These findings led the investigators to propose that radical quenching is the foundation for substrate-selective inhibition, as oxygenation of 2-AG is more highly dependent on peroxide tone than oxygenation of AA (24). Consistently, addition of 15-HPETE overcame fenamic acid–mediated inhibition of 2-AG oxygenation. The demonstration that 15-HPETE could also overcome the inhibition of 2-AG oxygenation by (*R*)-flurbiprofen suggests that this mechanism might extend to substrate-selective inhibitors of other classes. However, peroxide tone can theoretically play a role in the effectiveness of any inhibitor regardless of substrate, since slowing the COX reaction leads to a reduction in the formation of the hydroperoxide (PGG_2_ or PGG_2_-G), impeding enzyme activation. This effect is exacerbated under conditions of low peroxide tone.

Confirmation of substrate-selective inhibition by ibuprofen came through work by Dong *et al.* (42), who demonstrated that low concentrations of ibuprofen inhibit 2-AG by binding to *E*_allo_, to which, they argued, 2-AG does not bind. Inhibition curves for inhibition of AA oxygenation were found to be biphasic, suggesting competition between ibuprofen and AA for *E*_allo_ leading to mild inhibition followed by stronger inhibition resulting from competition with AA for *E*_cat_. They also showed that ibuprofen-mediated inhibition of 2-AG oxygenation was similar in native/native, Y385F/native, and Y385F/R120A dimer constructs. These results are consistent with the binding of ibuprofen in *E*_allo_ (the inactive Y385F monomer when present). However, a Y385F R120A/native heterodimer, which places both a catalytically inactivating mutation (Y385F) together with a mutation that eliminates ibuprofen binding (R120A) in the same monomer, retained sensitivity to ibuprofen-mediated inhibition of 2-AG oxygenation, although inhibitor potency was reduced. This finding suggests that ibuprofen must be capable of inhibiting 2-AG oxygenation by binding competitively to *E*_cat_ when binding to *E*_allo_ is not possible (42).

The discovery of substrate-selective inhibition of 2-AG oxygenation led to the hypothesis that it could be used *in vivo* to explore the role of PG-G biosynthesis without interfering with PG formation. Unfortunately, this goal has proven to be elusive. For example, Morgan *et al.* (48) detected PG-G biosynthesis in the brains of mice overexpressing PGHS-2 or following treatment with LPS. PG-G biosynthesis was blocked by lumiracoxib, (*R*)-flurbiprofen, and LM-4131, all of which had exhibited substrate-selective inhibition of 2-AG oxygenation *in vitro*. However, in all three cases, PG biosynthesis was also inhibited in the brain. In the case of (*R*)-flurbiprofen and LM-4131, these findings could be attributed to *in vivo* conversion to nonselective inhibitors (48). On the other hand, Khasabova *et al.* (49) reported the substrate-selective reduction in PGE_2_-G as compared with PGE_2_ levels in dorsal root ganglia isolated from a mouse model of sickle cell anemia following a 1 h pretreatment with 9 mg/kg (*R*)-flubiprofen. In this case, the short *in vivo* exposure may have minimized conversion of the (*R*)- to the (*S*)-enantiomer, thereby preserving substrate selectivity.

### Substrate-selective potentiation of 2-AG oxygenation

Consistent with the finding that nonsubstrate fatty acids stimulate the oxygenation of AA by PGHS-2, we discovered that 13-(*S*)-methylarachidonic acid (13-MeAA) selectively potentiates the PGHS-2-mediated oxygenation of 2-AG but not AA (50). Potentiation is attributed to an increase in *k*_cat_ and the prevention of substrate inhibition by blocking the binding of 2-AG to *E*_allo_. 13-Me-AA also restores the activity of PGHS-2 mutant enzymes that exhibit poor 2-AG oxygenating activity. It blocks the inhibition of 2-AG oxygenation by substrate-selective inhibitors and the inhibition of both AA and 2-AG by nonselective inhibitors. A crystal structure of 13-MeAA complexed with PGHS-2 revealed that it binds in the active site in an inverted conformation, with the carboxylic acid group penetrating deep into the active site channel and the hydrophobic tail at the constriction site at the entrance to the channel. A similar nonproductive conformation was observed in a crystal structure of AA bound to apo-PGHS-2 and in one subunit of AA bound to holo-PGHS-2 (20, 51). These observations suggest that inverted binding of fatty acids to one subunit (presumably *E*_allo_) could be a characteristic of allosteric potentiators. However, the crystal structure of palmitic acid complexed with PGHS-2 revealed binding of the fatty acid in only one subunit in a conventional orientation with the carboxyl group pointed toward the constriction (31).

## Production of PG-Gs in cells and *in vivo*

### Cell culture studies

The following discussion is limited to reports of PG-G formation from endogenous 2-AG, as those are the most relevant to the possible physiological or pathophysiological roles of these lipid mediators.

The first report of PG-G biosynthesis by cells in culture came from Kozak *et al.* (9). They pretreated RAW264.7 murine macrophage-like cells with LPS and interferon-γ for 7 h to induce PGHS-2 expression, followed by a 20 min exposure to ionomycin to trigger the production of 2-AG. The cells released 7.0 ± 0.1 ng/10^6^ cells of PGD_2_-G into the culture medium at the end of the incubation. PGD_2_-G formation was inhibited by the nonselective PGHS inhibitor indomethacin, the diacylglycerol (DAG) lipase inhibitor RHC-80267, and to a lesser extent by the phospholipase C (PLC) inhibitor U-73122. The latter results suggest a role for PLC-mediated hydrolysis of phosphatidylinositol 4,5-bisphosphate to produce AA-containing DAGs, followed by hydrolysis of those DAGs to provide the 2-AG substrate.

The discovery that murine resident peritoneal macrophages (RPMs) produce PG-Gs in response to a zymosan challenge was reported by Rouzer and Marnett (10). They observed the release of PGE_2_-G and PGI_2_-G (total of 16 ± 6 pmol/10^7^ cells) over a period of 2 h after zymosan addition. Synthesis of PG-Gs was substantially lower than that of the corresponding free acid PGs (PGE_2_ and PGI_2_, 21 ± 7 nmol/10^7^ cells). Pretreatment of the cells with LPS to induce PGHS-2 expression increased PG and PG-G synthesis but was not required, and the PGHS-2-selective inhibitor SC236 reduced PG-G and PG formation by 49% and 17%, respectively, in LPS-pretreated cells. The latter findings suggest an unanticipated role for PGHS-1 in PG-G formation in murine RPMs, which constitutively express high levels of that isoform. The ability of PGHS-1 to contribute to PG-G synthesis in zymosan-stimulated RPMs was further confirmed in studies using cells from mice bearing genetic deletions of the *Ptgs-1* or *Ptgs-2* genes (52).

In recognition of the importance of endocannabinoids in the nervous system, Duggan *et al.* (43) explored the ability of primary dorsal root ganglion (DRG) cells to produce PG-Gs. They demonstrated that pretreatment of the cells for 6 h with granulocyte–macrophage colony-stimulating factor, interferon-γ, and 15-HETE to induce PGHS-2 expression followed by a 3 h exposure to ionomycin resulted in the formation of PGE_2_-G and PGF_2α_-G along with their corresponding free acid and ethanolamide products. This was the first known report of the synthesis of PG-EAs by intact cells from endogenous substrate, though quantification of the various products was not provided (43).

Their interest in a possible role for PG-Gs in a malonate-mediated model of Huntington's disease led Valdeolivas *et al.* (53) to study PG-G biosynthesis in the M-213-2O striatal neuronal–like cell line. They found that a 6 h malonate treatment in the presence of the monoacylglycerol (MAG) lipase inhibitor OMDM169 induced PGHS-2 expression and led to the formation of PGE_2_-G (4 pmol/mg of lipid extract). Consistent with the Huntington's disease model, the malonate treatment was also toxic to the cells. The researchers were unable to detect PGE_2_-G in malonate-lesioned brain tissue from intact animals, even in the presence of MAG lipase inhibitors (53).

The discovery that inhibitors of alpha/beta-hydrolase domain–containing 6 (ABHD6, a hydrolase that hydrolyzes 2-AG) have anti-inflammatory effects in J774 macrophage–like cells, thioglycolate-elicited peritoneal macrophages, and BV2 microglial-like cells led Alhouayek *et al.* (54) to search for a mechanism. They found that ABHD6 inhibition increased 2-AG levels and that the observed anti-inflammatory effects were blocked by (*R*)-flurbiprofen. These observations suggest a possible role for PG-Gs, and, indeed, J774 cells produced PGD_2_-G (∼3 pmol/10^7^ cells) and PGE_2_-G (∼0.01 pmol/10^7^ cells) in response to an 8 h exposure to LPS. Synthesis of PGD_2_-G was blocked by HQL79, an inhibitor of PGD synthase (54). Similarly, Chicca *et al.* (46) exposed RAW264.7 cells to a combination of LPS, ATP, and thapsigargin for 8 h, leading to the formation of 300 to 400 pg PGE_2_-G/10^6^ cells, as compared with 40 ng of PGE_2_/10^6^ cells. In a recent study, Paquot *et al.* (55) used J774 cells treated with LPS for 24 h as a source of PG-Gs and PGs for the development of an LC–MS/MS assay. The cells produced PGD_2_-G in the highest concentration, followed by PGF_2α_-G, PGE_2_-G, and 15-deoxy-Δ^12,14^-PGJ_2_-G (a nonenzymatic dehydration product of PGD_2_-G) in that order. Levels of PG-Gs were approximately 100-fold lower than those of the corresponding free acid PGs. All analytes were detected in both the culture medium and cells, with greater abundance in the medium (55).

Recently, Aleem *et al.* (56) reported detailed time-course studies demonstrating that RAW264.7 cells treated with the chemically defined LPS analog Kdo2-lipid A (KLA) released PGs at high concentrations (∼1.2 nmol/10^6^ cells) over the first 12 h of exposure, followed by the release of PG-Gs (∼22 pmol/10^6^ cells) between 12 h and 24 h (56). This late-phase PG-G biosynthesis was accompanied by a gradual increase in 2-AG that started 6 h after KLA addition. The main products were PGD_2_-G and PGE_2_-G. The investigators further demonstrated that the RAW264.7 cells hydrolyzed PG-Gs with a half-life of 3 to 6 h, so detected levels underestimated total biosynthesis. PG-G biosynthesis was blocked by the DAG lipase inhibitor KT-172, which also reduced levels of 2-AG. Inclusion of the cPLA2 inhibitor giripladib during the KLA treatment eliminated the early KLA-mediated release of AA and blocked PG biosynthesis while modestly increasing 2-AG levels and having essentially no effect on PG-G formation. These findings rule out competition with AA as an explanation for the cells' failure to produce PG-Gs early in the KLA response, suggesting that the temporal dissociation of PG and PG-G biosynthesis is likely related to the differential availability of AA and 2-AG substrate pools to PGHS-2 over the course of the treatment (56).

### *In vivo* studies

The first report of PG-G detection *in vivo* came from Hu *et al.* (57), who identified PGE_2_-G in extracts of hind paw homogenates from rats that had not been treated previously with pharmacologic or inflammatory agents. PGE_2_-G was detected at levels of fmol/paw as compared with PGE_2_, which was present at pmol/paw levels. Injection of carrageenan into the paws to induce inflammation resulted in increased PGE_2_ but not PGE_2_-G levels. Formation of PGE_2_-G in rat hind paws was partially blocked by inhibitors of DAG lipase and PGHS (nonselective). The investigators further demonstrated that PGE_2_-G was rapidly hydrolyzed in hind paw tissue, suggesting that the observed low levels could well underestimate total formation of the eicosanoid.

In their examination of the role of 2-AG and PG-Gs in colon health and disease, Alhouayek *et al.* (58) demonstrated the presence of 15-deoxy-Δ^12,14^-PGJ_2_-G, presumably derived from PGD_2_-G, in mouse colon tissue. Levels of the compound were decreased in colons from mice treated with dextran sodium sulfate (DSS) to induce colitis (58).

Khasabova *et al.* (49) used the murine HbSS-BERK model of sickle cell disease to study the origins of neuropathic pain. They discovered that PGHS-2 expression was increased in DRGs from HbSS mice as compared with HbAA control mice. Analysis of lipid extracts of the DRGs from both HbSS and control mice revealed the presence of PGE_2_-G in both but at much higher levels in those from HbSS mice (49).

Having developed their assay for PG-G analysis mentioned above, Paquot *et al.* (55) used it to search for PGE_2_-G in a number of tissues from DSS-treated mice *versus* controls. PGE_2_-G was detected in the colon, visceral adipose tissue, lymph nodes, and spleen. Levels in mice with colitis were elevated in adipose tissue, reduced in the colon, and unchanged in lymph nodes and spleen when compared with those from control mice (55).

## Biological effects of PG-Gs

### PGE_2_-G

Despite the fact that PG-Gs were described 25 years ago, our understanding of their physiological or pathophysiological importance is incomplete. Nirodi *et al.* (59) first reported that PGE_2_-G causes Ca^2+^ mobilization in RAW264.7 cells at picomolar to nanomolar concentrations (59). PGE_2_-G was not hydrolyzed to PGE_2_ during the time course of treatment, and PGE_2_ was inactive at the same and higher concentrations. Ca^2+^ mobilization was associated with an increase in inositol 1,4,5-trisphosphate (IP_3_) concentrations, and PKC was activated by PGE_2_-G, suggesting the production of DAG. Furthermore, PGE_2_-G stimulated extracellular signal–regulated kinase (ERK) phosphorylation in a concentration-dependent fashion. PKC activation and ERK phosphorylation were blocked by a PLC inhibitor, an IP_3_ receptor antagonist, and a PKC inhibitor. These observations suggest that PGE_2_-G binds to a receptor, triggering PLC hydrolysis of phosphatidylinositol 4,5-bisphosphate to IP_3_ and DAG. Studies with recombinant eicosanoid G protein–coupled receptors have shown that PGE_2_-G does not bind strongly to any of the PG or Tx receptors, in particular to the PGE_2_ receptors, EP1, EP2, EP3, and EP4. Subsequent studies with the human lung carcinoma cell line H1819 revealed a more robust mobilization of Ca^2+^ by PGE_2_-G and structural analogs that contain a nonhydrolyzable amide linkage in place of the ester oxygen. PGE_2_-G and the amide analogs displayed activity at picomolar concentrations and exhibited bell-shaped concentration dependences with less activity at higher concentrations (60).

The Chen laboratory reported that the addition of PGE_2_-G to primary cultured mouse hippocampal neurons increases the frequency but not the amplitude of miniature inhibitory postsynaptic currents (61). The effect of PGE_2_-G was not blocked by a CB1 receptor antagonist but was blocked by an IP_3_ receptor antagonist and a mitogen-activated protein (MAP) kinase inhibitor but not by a PKC inhibitor. PGE_2_-G was more active than PGD_2_-G or PGF_2α_-G. These effects are the opposite of those reported for 2-AG, which decreases the frequency of miniature inhibitory postsynaptic currents (61). As reported elsewhere, inhibition of PGHS-2 augments the depolarization-induced suppression of inhibition triggered by 2-AG, whereas induction of PGHS-2 abolishes depolarization-induced suppression of inhibition (62). The latter observations suggest that PGHS-2 may alter the levels of 2-AG by converting it to PG-Gs that exhibit opposite effects on neuronal transmission.

Subsequent work from the Chen laboratory demonstrated that PGE_2_-G enhances the frequency but not the amplitude of miniature excitatory postsynaptic currents in mouse hippocampal neurons (63). The effect was not dependent on the CB1 receptor but was significantly attenuated by an IP_3_ receptor antagonist as well as a p38 MAP kinase inhibitor and an ERK inhibitor. The increase in the frequency of miniature excitatory postsynaptic currents is likely because of enhanced release of presynaptic glutamate, which leads to an increase in neurotoxicity through the *N*-methyl-d-aspartate receptor. The increase in the frequency of postsynaptic currents induced by PGE_2_-G is mediated by an unknown receptor(s) that enhances the release of gamma-aminobutyric acid (GABA) in the case of inhibitory currents and glutamate in the case of excitatory currents.

Building on these observations, Yang *et al.* (64) reported that PGE_2_-G, and to a lesser extent PGD_2_-G and PGF_2α_-G, elevate long-term potentiation (LTP) in mouse hippocampal slices. PGE_2_-G-mediated enhancement of LTP was inhibited by inclusion in the bath solution of an IP_3_ receptor antagonist, a p38 MAP kinase inhibitor, or an ERK inhibitor. PGE_2_-G treatment led to phosphorylation of both p38 MAP kinase and ERK 1/2. PGE_2_-G may contribute to the enhancement of LTP observed in mice treated with LPS, which strongly induces PGHS-2 in the brain. However, it should be noted that PGHS-2 induction produces PGE_2_ from AA, which can enhance LTP, and it decreases the levels of 2-AG (which inhibits LTP) through oxidative metabolism. Thus, the mechanism of PGHS-2's contribution to LTP appears to be complex. Nevertheless, there is a consistent pattern in these experiments demonstrating that PGE_2_-G binds to non-EP receptors to trigger an increase in intracellular IP_3_ and DAG. The former binds to its receptor that elevates intracellular Ca^2+^ and activates MAP kinase signaling to enhance synaptic transmission.

As stated above, Hu *et al.* (57) first reported the isolation of PGE_2_-G in an intact animal, the rat. They also reported the first *in vivo* effects of PGE_2_-G—the induction of thermal hyperalgesia and mechanical allodynia. By using a cocktail of EP1–4 receptor antagonists, they were able to demonstrate that some of these effects are due to PGE_2_ formed by hydrolysis of PGE_2_-G. However, in contrast to the inhibitory effects of these compounds on thermal hyperalgesia and mechanical allodynia induced by PGE_2_, they did not completely inhibit the effects of PGE_2_-G, consistent with the existence of a separate receptor that mediates the effects of PGE_2_-G (57).

Lindgren *et al.* (65) discovered a role for PGHS-2 oxygenation of 2-AG in modulating synaptic signaling in the neuromuscular junction. Activation of the muscarinic receptor is known to stimulate the release of 2-AG from the postsynaptic muscle cell, which activates the CB1 receptor on the presynaptic neuron and desensitizes the neuron for further neurotransmitter release (62, 66, 67). However, approximately 30 min after muscarinic stimulation, the neuromuscular junction exhibits enhanced acetylcholine release and signaling. This enhancement is associated with an induction of PGHS-2 synthesis in adjacent glial cells, in this case, perisynaptic Schwann cells. In the studies of Lindgren *et al.* (65), treatment with PGHS-2 inhibitors enhanced 2-AG-mediated desensitization but inhibited subsequent enhancement. Application of PGE_2_-G to the neuromuscular junction caused a large and persistent increase in end-point potential amplitude, characteristic of enhanced acetylcholine release. Such an increase was not observed when PGE_2_ was administered. PGE_2_-G enhanced the frequency of neurotransmitter release but not the sensitivity of the postsynaptic acetylcholine receptors, suggesting that its action is presynaptic. Interestingly, the stimulatory effect of PGE_2_-G was not blocked by the EP1/2 receptor antagonist, AH6809, but was completely blocked by capsazepine, an antagonist for transient receptor potential cation channel subfamily V member 1 and transient receptor potential cation channel subfamily M member 8 (65). These observations are analogous to those from the Chen laboratory demonstrating that PGE_2_-G can act presynaptically to enhance release of the inhibitory neurotransmitter, GABA, or the excitatory neurotransmitter, glutamate.

As discussed above, Khasabova *et al.* (49) reported the induction of PGHS-2 in the DRG of HbSS-BERK mice, which are a model for sickle cell–induced pain. The levels of PGE_2_-G and PGE_2_ were elevated in the HbSS-BERK DRGs but not in DRGs from HbAA-BERK mice, which do not develop sickle cell disease. Treatment of the mice with the substrate-selective inhibitor, (*R*)-flurbiprofen, reduced the levels of PGE_2_-G but not PGE_2_. It also decreased mechanical and thermal hyperalgesia and decreased sensitization of nociceptors in HbSS-BERK mice. Intraplantar injection of PGE_2_-G into the paws of control HbAA-BERK mice induced sensitization of nociceptors and hyperalgesia. A combination of antagonists for the EP receptors activated by PGE_2_ did not block the mechanical hyperalgesia induced by PGE_2_-G. These findings indicate PGE_2_-G, generated by PGHS-2 oxygenation of 2-AG, is a mediator of sickle cell–induced pain, that its formation can be selectively blocked with (*R*)-flurbiprofen, and that its actions are distinct from those induced by PGE_2_.

The identity of the receptor in RAW264.7 cells responsible for the actions of PGE_2_-G was the basis of a study by Brüser *et al.* (68). They profiled the G protein–coupled receptors expressed in cells that are responsive and nonresponsive to PGE_2_-G and found that expression of the nucleotide receptor P2Y_6_ correlated to responsiveness in the cells. siRNA knockdown of P2Y_6_ eliminated responses to PGE_2_-G, and expression of P2Y_6_ in human embryonic kidney 293 cells rendered the cells responsive to PGE_2_-G. Recent reports using molecular modeling and mutagenesis of residues in the putative ligand-binding site indicated that the PGE_2_-G-binding site partially overlaps the binding site of UDP, the cognate ligand for P2Y_6_ (68, 69). Interestingly, the P2Y_6_ antagonist MRS2578 abolished mechanical and thermal hyperalgesia induced by PGE_2_-G in HbSS-BERK mice (49). Whether P2Y_6_ is responsible for the ability of PGE_2_-G to stimulate presynaptic GABA or glutamate release has not been tested.

### PGD_2_-G and its dehydration products

Alhouayek *et al.* (54) demonstrated that treatment of J774 macrophages with an ABHD6 inhibitor, WWL70, increases the levels of 2-AG and inhibits the synthesis of IL-1β mRNA following activation by LPS. This anti-inflammatory effect was neither blocked by antagonists of CB1 or CB2 nor did CB1 or CB2 agonists mimic the effect of WWL70, indicating that 2-AG is not directly responsible. The substrate-selective PGHS-2 inhibitor, (*R*)-flurbiprofen, inhibited the effect of WWL70, as did the PGD synthase inhibitor, HQL79. This implies that the anti-inflammatory effect of WWL70 is due to PGHS-2 oxidation of 2-AG to PGD_2_-G and, indeed, PGD_2_-G alone inhibits IL-1β mRNA induction in J774 cells treated with LPS. As noted above, the biosynthesis of PGD_2_-G by LPS-activated J774 cells was verified by LC–MS/MS as was the ability of HQL79 to selectively inhibit its synthesis. The use of antagonists for the PGD_2_ receptors, DP1 and DP2, and the nuclear receptors, peroxisome proliferator–activated receptors, PPARα and PPARγ, indicate that these receptors are not responsible for the anti-inflammatory effect of PGD_2_-G. PGD_2_-G administration to C57Bl6 mice reduced the synthesis of proinflammatory cytokines in the cerebellum and liver induced by systemic treatment with LPS (54). The *in vivo* effects of PGD_2_-G are complicated by its potential hydrolysis to PGD_2_ as well as its dehydration to 15-deoxy-Δ^12,14^-PGJ_2_-G.

More recently, these results were confirmed and extended by Scheaffer *et al.* (70), who reported that PGD_2_-G attenuated the induction of IL-6 and IL-1β mRNA by murine RPMs treated with LPS. The anti-inflammatory effect of PGD_2_-G was enhanced by inhibitors of carboxylesterase-1. Similar results were observed with human THP-1 macrophages. Carboxylesterase-1 is more abundant in THP-1 cells than either MAG lipase or ABHD6 and accounts for more than 50% of the PGD_2_-G hydrolase activity in the cells.

Further work by Alhouayek *et al.* (58) showed that intraperitoneal administration of PGD_2_-G to mice treated with DSS reduces the severity of colitis induced by DSS. PGD_2_-G treatment was accompanied by significant reductions in several markers of inflammation, including IL-1β, TNFα, IL-6, MIP-1α, iNOS, PGHS-2, MMP-2, and MMP-9. Some of the anti-inflammatory effects were seen if PGD_2_-G was administered 5 days after DSS treatment. Similar effects were not observed when the mice were treated with PGD_2_ or PGD_2_-EA. PGD_2_-G was shown to bind to the DP1 receptor with equal potency to PGD_2_ but with 10-fold less potency to DP2. A DP1 agonist also exhibited anti-inflammatory effects in the colitis model, and a DP1 antagonist blocked the effects of PGD_2_-G. PGD_2_-G was not detected basally in colon tissue but 15-deoxy-Δ^12,14^-PGJ_2_-G was (58). 15-deoxy-Δ^12,14^-PGJ_2_-G has been reported to activate the PPARγ receptor (71), and in the DSS colitis model, PPARγ antagonists partially blocked the effects of PGD_2_-G (58). Thus, in contrast to the results reported in J774 macrophages, the *in vivo* effects of PGD_2_-G appear to be mediated by DP1 and PPARγ. Since PGD_2_-G does not activate PPARγ, this receptor appears to be activated by its dehydration product, 15-deoxy-Δ^12,14^-PGJ_2_-G (58, 71).

Buisseret *et al.* (72) reported the inhibition of carrageenan-induced edema and hyperalgesia in the mouse paw by PGD_2_-G. PGD_2_-G also inhibited the production of certain inflammatory markers (*e.g.*, iNOS, PGHS-2, TNFα, IL-6) in the carrageenan model. PGD_2_ was inactive. Interestingly, in contrast to its impact on DSS-induced colitis, PGD_2_-G action was not blocked by an antagonist of the DP1 receptor. Also, in contrast to the colitis model, 15-deoxy-Δ^12,14^-PGJ_2_-G did not inhibit hyperalgesia in the carrageenan model. PGD_2_-G inhibited hyperalgesia in the LPS-induced mouse foot-pad model, but the extent of reduction was not as significant as observed in the carrageenan model (73).

Recently, Mwema *et al.* (74) explored the potential of encapsulating PGD_2_-G in lipid nanocapsules for delivery *via* the nose-to-brain method. This is an approach to circumvent the rapid hydrolysis of PGD_2_-G to PGD_2_ that occurs in blood, especially of rodents, following systemic administration (75). PGD_2_-G was stable in the nanocapsules for up to 14 days, and when PGD_2_-G-containing nanocapsules were applied to the apical surface of a monolayer of primary rat olfactory cells, material emerging from the basolateral surface of the cells within 4 h was capable of reducing inflammatory gene expression when applied to BV2 microglia cells (74). Furthermore, PGD_2_-G was identified by LC–MS/MS in the basolateral material. Lipid nanocapsules containing PGD_2_-G were detectable in the brains of mice following intranasal installation. Brain levels were increased by adding the cell-penetrating protein, TAT, to the PGD_2_-G-containing nanocapsules. Anti-inflammatory activity was observed in the olfactory bulb, prefrontal cortex, brainstem, and spinal cord following intranasal administration, but the interpretation is complicated by the observation that lipid nanocapsules alone, without PGD_2_-G, exhibited the ability to reduce expression of some but not all markers of inflammation. PGD_2_-G-containing lipid nanoparticles did not have a positive impact on demyelination or remyelination of the spinal cords of mice in the experimental autoimmune encephalitis model of multiple sclerosis (74, 76). Thus, although this novel drug delivery method is promising, additional experimentation is necessary to optimize it to deliver PGD_2_-G as an anti-inflammatory agent.

### What do we know; what do we not know?

As described above, PGHS-2 oxidizes a broad range of substrates, including ester, amide, and lysophospholipid derivatives of AA. The efficiency of oxygenation varies significantly, but 2-AG appears to be the preferred substrate. 2-AG is also the most abundant of these molecules, many of which are minor constituents found in the brain (77, 78). The productive binding pose of 2-AG in the PGHS-2 active site is similar to that of AA, although subtle differences in the orientation of the 13-*pro*(*S*)-hydrogen may render 2-AG more sensitive than AA to allosteric inhibitors or potentiators and to agents that lower peroxide tone.

Oxygenation of 2-AG to PG-Gs occurs in cells and in intact animals in response to physiological or pathophysiological stimuli. These are the same stimuli that generate PGs from AA even though the signaling pathways and enzymes that release 2-AG and AA are different. No stimuli have yet been found that selectively release 2-AG in preference to AA, and rapid *in vivo* hydrolysis of 2-AG to AA by cellular esterases (79, 80, 81, 82) complicates identifying such a stimulus.

The amounts of PG-Gs detected in cells and *in vivo* are much lower than the amounts of PGs. Several factors are responsible for this. The first is that the pool sizes of AA released by PLA2 activation are higher than the pool sizes of 2-AG released by PLC; in macrophages, the ratio is ∼10 to 50 to one under conditions that have been studied thus far (10, 52, 56). The second is that if AA and 2-AG are released simultaneously, AA is a better substrate for PGHS-2 because of allosteric modulation. AA *inhibits* the oxygenation of 2-AG, whereas 2-AG *activates* the oxygenation of AA (41). Another factor is that hydrolysis of PG-Gs causes an underestimate of their actual levels (83). This is because the products of hydrolysis are not unique metabolites but rather PGs. In contrast, the AA metabolites TxA_2_ and PGI_2_ are also extremely unstable to hydrolysis and have never been directly quantified in cells or tissues, but the products of their hydrolysis are the unique metabolites TxB_2_ and 6-keto-PGF_1α_ (84, 85). That is not the case for PG-Gs.

As noted above, 2-AG also undergoes rapid hydrolysis in cells and *in vivo* (79, 80, 81, 82). This is an important mechanism for reducing endocannabinoid tone because its hydrolysis product, AA, does not activate the cannabinoid (CB) receptors, CB1 or CB2. So, it is often challenging to measure the production of 2-AG, especially in real time. The recent development of genetically encoded sensors (86) allows real-time detection of signaling molecules by fluorescence methods, and indeed, such a sensor has been described for 2-AG (87). It would be very attractive to develop a similar sensor for PG-Gs, although it will require either the identification or engineering of a high sensitivity/selectivity receptor that can be fused to GFP derivatives. Given the very low levels of PG-Gs detected in many cellular experiments, it is not clear if a sensor could be developed that would generate a sufficient fluorescence signal on PG-G binding to be detected.

Aleem *et al.* (56) have demonstrated a lag in the time courses for the production of 2-AG and PG-Gs relative to those for AA and PGs in RAW264.7 macrophages following activation with KLA. This suggests a novel pathway in these cells that will need to be the focus of detailed investigations of the role of various lipase enzymes in the release of 2-AG. Genetic approaches or degron approaches should be employed to define the sequence by which 2-AG is generated. Similar approaches could be used to better define the identity of the hydrolases that convert PG-Gs to PGs in specific cells and tissues. Multiple enzymes have been reported to hydrolyze PG-Gs, but their individual contributions to PG-G modulation *in vivo* remain unclear (70, 75, 88, 89, 90, 91).

Tools with which to dissect the role of PG-Gs as signaling agents are lacking, so it has been difficult to link a specific biological effect to the production of a particular PG-G. The best example of defining a role for endogenously produced PG-Gs is the work of Alhouayek *et al.* (54) demonstrating that PGD_2_-G produced by J774 macrophages inhibits cytokine production. They used a combination of the substrate-selective inhibitor, (*R*)-flurbiprofen, and the PGD synthase inhibitor, HQL79, to enhance the immune response to LPS while blocking the production of PGD_2_-G. Although (*R*)-flurbiprofen was useful in this setting, it and other substrate-selective inhibitors have not proven to be generally useful in intact animals because of their metabolism *in vivo*.

No specific receptors for PG-Gs have been identified. PGE_2_-G binds poorly to the EP receptors for PGE_2_, but it has been described as a high potency ligand for the P2Y_6_ receptor (59, 68, 69). In fact, it is 10^5^-fold more potent than the cognate ligand, UDP (68). This correlates to the picomolar potency of PGE_2_-G at activating Ca^2+^ mobilization in RAW264.7 cells (59). However, activated macrophages, including RAW264.7 cells, release UDP at micromolar concentrations following LPS activation, so it is difficult to dissect the contributions of PGE_2_-G from those of UDP to a P2Y_6_-driven response (92, 93). PGE_2_-G may also activate the transient receptor channels, transient receptor potential cation channel subfamily V member and transient receptor potential cation channel subfamily M member 8, but again it is not a unique ligand for these channels (65).

PGD_2_-G binds as well as PGD_2_ to the DP1 receptor but less well to the DP2 receptor (58). Binding to DP1 may account for PGD_2_-G's anti-inflammatory activity in some models but not others. PGD_2_-G does not activate the nuclear receptor, PPARγ, but its dehydration product, 15-deoxy-Δ^12,14^-PGJ_2_-G, does (71, 94). So PGD_2_-G may indirectly activate PPARγ by serving as a precursor to 15-deoxy-Δ^12,14^-PGJ_2_-G.

**Figure 6:**
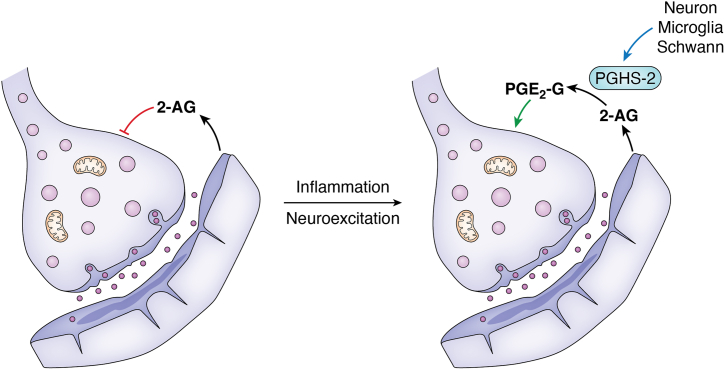
**Potential role of PGHS-2-mediated 2-AG oxygenation in neuronal signaling.** 2-AG produced by postsynaptic cells binds to CB1 receptors in presynaptic neurons, resulting in depressed neurotransmitter release. Induction of PGHS-2 in microglia, neurons, or Schwann cells can lower CB tone by oxygenating 2-AG to produce PGE_2_-G, which then increases neuron firing, thereby reinforcing long-term potentiation. 2-AG, 2-arachidonoylglycerol; CB, cannabinoid; PGHS, prostaglandin endoperoxide synthase.

The most likely sites of importance of PGHS-2-catalyzed oxygenation of 2-AG are the immune system and the nervous system. 2-AG is active in both settings, and PG-Gs demonstrate either reinforcing or opposing effects. PGD_2_-G is the major 2-AG metabolite in several macrophage cell lines (54, 56), and it exhibits anti-inflammatory activity in multiple models (54, 58, 72). PGE_2_-G is a major metabolite in the nervous system, where it induces thermal hyperalgesia and mechanical allodynia (57). Several studies have demonstrated that PGE_2_-G induces the release of neurotransmitters from presynaptic neurons, increasing the frequency of inhibitory or excitatory transmission (61, 63, 65). This is associated with an increase in LTP (64). PGHS-2 exists basally in some neurons but is induced in other populations of neurons as well as microglia and Schwann cells by inflammatory stimuli or neuronal stimulation (54, 65, 95). 2-AG released from postsynaptic neurons plays an important role in modulating neuronal transmission by binding to CB1 receptors on presynaptic neurons to deactivate transmission. The induction of PGHS-2 lowers CB tone by oxygenation of 2-AG and increases neurotransmission by the production of PGE_2_-G (Fig. 6).

A comprehensive picture of the biological consequences of PGHS-2 oxygenation of 2-AG is not currently available, but as the old saying goes, “Where there's smoke, there's fire.” The available data suggest that PGHS-2-dependent metabolism of 2-AG to PG-Gs has an important place in the modulation of inflammation and brain function, but additional experiments will be required to establish it.

## Dedications

This article is part of a special issue honoring the memory of William L. Smith. We dedicate this article to him. Bill was an exceptional scientist who made seminal contributions to every aspect of our understanding of the oxygenation of arachidonic acid by prostaglandin H synthase. He possessed a quick intellect and the ability to identify important problems as well as design definitive experiments to solve them. He also gave his time generously to the scientific community as department chair at Michigan State University and the University of Michigan as well as Associate Editor of the *Journal of Biological Chemistry*. His success in these roles was facilitated by his outstanding judgment and wry sense of humor. He was a long-time friend and colleague.

## Conflict of interest

The authors declare that they have no conflicts of interest with the contents of this article.
